# Relationships between pathologic subjective halitosis, olfactory reference syndrome, and social anxiety in young Japanese women

**DOI:** 10.1186/s40359-017-0176-1

**Published:** 2017-03-14

**Authors:** Miho Tsuruta, Toru Takahashi, Miki Tokunaga, Masanori Iwasaki, Shota Kataoka, Satoko Kakuta, Inho Soh, Shuji Awano, Hiromi Hirata, Masaharu Kagawa, Toshihiro Ansai

**Affiliations:** 1 Division of Community Oral Health Development, Kyushu Dental University, 2-6-1 Manazuru, Kokurakita-ku, Kitakyushu, 803-8580 Japan; 2 Graduate School of Human Environment Science, Fukuoka Women’s University, Fukuoka, Japan; 3 School of Nutrition & Dietetics, Kanagawa University of Human Services, Yokosuka, Japan; 4 Division of Clinical Education Development and Research, Kyushu Dental University, Kitakyushu, Japan; 5 Developmental Clinical Psychology, Faculty of Nutrition, Kagawa Nutrition University, Sakado, Japan; 6 Institution of Nutrition Sciences, Kagawa Nutrition University, Sakado, Japan

**Keywords:** Pathologic subjective halitosis, Olfactory reference syndrome, Social anxiety, Pseudohalitosis, Brief psychotherapy, Health volunteers

## Abstract

**Background:**

Pathologic subjective halitosis is known as a halitosis complaint without objective confirmation of halitosis by others or by halitometer measurements; it has been reported to be associated with social anxiety disorder. Olfactory reference syndrome is a preoccupation with the false belief that one emits a foul and offensive body odor. Generally, patients with olfactory reference syndrome are concerned with multiple body parts. However, the mouth is known to be the most common source of body odor for those with olfactory reference syndrome, which could imply that the two conditions share similar features. Therefore, we investigated potential causal relationships among pathologic subjective halitosis, olfactory reference syndrome, social anxiety, and preoccupations with body part odors.

**Methods:**

A total of 1360 female students (mean age 19.6 ± 1.1 years) answered a self-administered questionnaire regarding pathologic subjective halitosis, olfactory reference syndrome, social anxiety, and preoccupation with odors of body parts such as mouth, body, armpits, and feet. The scale for pathologic subjective halitosis followed that developed by Tsunoda et al.; participants were divided into three groups based on their scores (i.e., levels of pathologic subjective halitosis). A Bayesian network was used to analyze causal relationships between pathologic subjective halitosis, olfactory reference syndrome, social anxiety, and preoccupations with body part odors.

**Results:**

We found statistically significant differences in the results for olfactory reference syndrome and social anxiety among the various levels of pathologic subjective halitosis (*P* < 0.001). Residual analyses indicated that students with severe levels of pathologic subjective halitosis showed greater preoccupations with mouth and body odors (*P* < 0.05). Bayesian network analysis showed that social anxiety directly influenced pathologic subjective halitosis and olfactory reference syndrome. Preoccupations with mouth and body odors also influenced pathologic subjective halitosis.

**Conclusions:**

Social anxiety may be a causal factor of pathologic subjective halitosis and olfactory reference syndrome.

## Background

Pathologic halitosis is defined as the existence of a preoccupation with unpleasant mouth odor [1] and has different causes that may originate in various bodily locations such as the oral cavity, nasal cavity, upper respiratory tract, and lungs [2]. According to a report by Aydin et al. [3], pathologic halitosis has been etiologically classified into five types: oral, airway, gastroesophageal, blood-borne, and pathologic subjective halitosis. Pathologic subjective halitosis (Type 5) is known as a halitosis complaint without objective confirmation by others or by halitometer measurement [3]. That is, the patient believes there is halitosis, but no odor is clinically detectable [4].

Pathologic subjective halitosis can be divided into two types: one with psychologic and the other with neurologic origins. The former includes obsessive-compulsive spectrum disorder and olfactory reference syndrome; the latter includes several chemosensory disorders such as olfaction and gustation [3]. Most pathologic subjective halitosis complaints are attributed to psychological factors, but at least some are neurological [3]. To date, it has been reported that 75% of olfactory reference syndrome patients present with pathologic subjective halitosis complaints [5]. Olfactory reference syndrome, descriptions of which have existed for over a century [5], is a preoccupation with the false belief that one emits a foul or offensive body odor. This syndrome has been defined as a psychiatric condition characterized by a persistent preoccupation with body odor accompanied by shame, embarrassment, significant distress, avoidance behavior, and social isolation [6]. Typically, patients with this condition are preoccupied with concerns about multiple body parts [5, 7]. Of these concerns, mouth odor is the most common source of the preoccupation [5]. Olfactory reference syndrome may present in patients with obsessive-compulsive disorder or social anxiety disorder, and has reportedly been more prevalent in social anxiety disorder than in obsessive-compulsive disorder [8].

The association between pathologic subjective halitosis and psychological variables has been reported previously [9]. All psychological conditions including depression, anxiety, and stress demonstrated a significant association with subjective pathologic halitosis, and anxiety seemed to be the greatest risk factor [10]. Recently, Zaitsu et al. reported that patients with pseudohalitosis are at risk for social anxiety disorder [11]. Pseudohalitosis is a category of halitosis in which obvious malodor is not perceived by others, but the patient insists that it exists [12]; this is also categorized as pathologic subjective halitosis [3]. Social anxiety is defined as anxiety resulting from the prospect or presence of personal evaluation in a real or imagined social situation [13]. Patients with olfactory reference syndrome may experience symptoms similar to those of pathologic subjective halitosis [3].

Different studies have been reported around the world regarding the prevalence rate of pathologic subjective halitosis. In a systematic review conducted in the USA and Brazil, the prevalence rate varied from 22 to 32% [14]. Moreover, controversy concerning the effect of gender on pathologic subjective halitosis still remains. For instance, several studies reported that the prevalence of pathologic subjective halitosis is higher in females [15], while no significant differences in gender have been found [10]. Furthermore, as previously described in several reports, young people reported to be suffering more from pathologic subjective halitosis than older individuals [10].

Several studies examining dental patients suggested that females were more anxious about mouth odor and tended to perceive that they had oral malodor [16]. To date, little is known about the causal relationships between social anxiety and pathological conditions such as pathologic subjective halitosis and olfactory reference syndrome. Therefore, in the present study, considering the difference of gender on potential psychological connections existing in those relationships, we performed a large-scale epidemiological survey focusing on females. We used Bayesian network principles in the analysis of potential causal relationships between pathologic subjective halitosis, olfactory reference syndrome, social anxiety, and preoccupations with body part odors (e.g., body, foot, mouth, and armpit).

## Methods

### Participants

This was a cross-sectional study of pathologic subjective halitosis conducted in Japan. The proportion of pathologic subjective halitosis in the general population is thought to be small, because a previous study that used organoleptic evaluation and questionnaire survey regarding halitosis in a health examination for 153 community-dwelling adults (75 men and 78 women, mean age: 50.3 years) found that the percentage of those who believed that they had bad mouth odor but had no serious mouth odor detected was about 8% [17]. Hence, we considered that a large number of participants was needed to accurately elucidate the features of pathologic subjective halitosis. Preoccupation with mouth odor has been found to be prevalent in young females around 20 years old [18]. Another survey conducted in Japan reported that more females were concerned about their mouth odor than males, and prevalence rates among females were higher in younger populations (teens or twenties) [19]. Therefore, we focused on a population of community-dwelling female students in this study.

We initially asked professors, associate professors, and lecturers from academic departments in 13 colleges and universities located in Fukuoka, Hyogo, Osaka, Kyoto, Aichi, Shizuoka, Kanagawa, and Tokyo prefectures to recruit the study participants, who were 1640 student volunteers attending classes related to health science. The departments were Food Science, Nutrition, Culinary, Dietitian, Dental Hygiene, Food Culture, French Literature, International Studies, Sociology, Children’s Health, Occupational Therapy, and Physical Therapy.

**Fig. 1:**
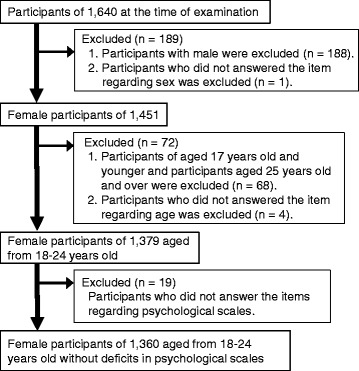
Flow diagram of participant selection

The final number of eligible participants was 1360, all of whom were females aged 18 – 24 years old, with a mean (±SD) age of 19.6 ± 1.1 years (Fig. 1).

### Exclusion criteria

Missing items regarding sex and men (*n* = 189) were excluded, because the present study focused on females. Further, participants younger than 17 and older than 25 years, or with unreported ages (*n* = 72) were excluded because we focused on university students. Missing items regarding psychological scales were also excluded because of problems with reliability (*n* = 19).

### Procedure

This study was conducted in accordance with guidelines laid down in the Declaration of Helsinki and approved by the Ethics Committee of Kyushu Dental University (No. 13–70). A paper-based survey questionnaire was distributed during classroom sessions regarding health science at each participating college or university. The participants were explained the nature of the research project and provided written informed consent prior to completion. There was no incentive given for participation in this study.

### Questionnaire

We employed a self-administered questionnaire consisting of items regarding sex and age, as well as the scale for pathologic subjective halitosis reported by Tsunoda et al. [20], a scale for olfactory reference syndrome [21], and a scale for social anxiety [22].

### Pathologic subjective halitosis

We used the original scale of Tsunoda et al. that was developed as a tool to screen for the extent of pathologic subjective halitosis, which consists of 10 items used to rate the intensity of the belief in emitting mouth odor, delusion of reference, and disturbance of social adaptation [20]. The items were scored on a 3-, 4-, or 5-point scale, with scores ranging from 10 to 45. The normal group included scores from 10 to 13, the moderate group scores from 14 to 21, and the severe group scores of 22 and higher. Representative examples of the items include, “How strong is your mouth odor,” “Does your family notice your bad breath?”, and “Have you ever heard someone talking about your mouth odor with others rather than telling you?” (translated from Japanese). Higher scores represent a greater tendency for pathologic subjective halitosis.

### Preoccupations with body part odor

We also asked about preoccupation with odor from various body parts, for example, “Which body parts do you care about for etiquette?” (translated from Japanese). Participants were allowed to choose one or more from mouth, body, armpit, and foot [5, 7].

### Olfactory reference syndrome

Olfactory reference syndrome was examined using a scale constructed for a study performed in Japan that included 4 conditions: 1) odor leakage from the body, 2) my odor gives discomfort to others, 3) I am avoided by others because of my odor, and 4) others consider me to be dirty because of my odor [21]. The scale consists of 7 items scored on a 5–point scale from 1 (not at all characteristic or true of me) to 5 (extremely characteristic or true of me), with total scores ranging from 7 to 35. Sample items include “my body odor makes others uncomfortable,” “I sometimes sense a strange odor emanating from my body,” and “I occasionally think that I am disliked by others because of my smell” (translated from Japanese). Higher scores were considered to represent a greater tendency for olfactory reference syndrome.

### Social anxiety

The original scale for social anxiety was developed by Leary in 1983 [13]. In the present study, we used the Japanese version constructed in 1991 [22], which is based on the original social anxiety scale. The scale consists of 7 items scored on a 5-point scale ranging from 1 (no tendency at all) to 5 (extremely characteristic or true of me), with the total score for social anxiety ranging from 7 to 35 [22]. A sample item is “I usually feel uncomfortable when I am in a group of people I don’t know.” Higher scores indicate a greater tendency for social anxiety.

### Statistical analyses

Data analysis was performed using SPSS statistics version 20 (IBM Japan Ltd, Tokyo, Japan) and R version 3.1.1 (the R Project for Statistical Computing, Vienna, Austria). A probability of less than 0.05 was used to indicate statistical significance.

Participants were divided into groups based on the pathologic subjective halitosis scale: the normal group included scores of 10 – 13; the moderate group included scores of 14 – 21; and the severe group included scores of 22 and higher.

Differences based on the pathologic subjective halitosis scale between the three groups in social anxiety and olfactory reference syndrome were analyzed using the Kruskal-Wallis test. The relationships between pathologic subjective halitosis and preoccupations with odors of body parts such as mouth odor, body odor, armpit odor, and foot odor, were analyzed using a chi-squared test. Residual analysis was used to indicate higher or lower incidence compared to expected values in each group of variables when the chi-squared test was significant.

We employed a Bayesian network to examine potential causations between pathologic subjective halitosis, olfactory reference syndrome, social anxiety, and preoccupations with body part odors (e.g., body, foot, mouth, and armpit). A Bayesian network can indicate causal relationships using Bayes’ theorem between variables without the authors’ prejudice affecting data [23].

## Results

Table 1 shows median values, along with the 25^th^ and 75^th^ percentiles and range of scales for pathologic subjective halitosis, olfactory reference syndrome, and social anxiety, as well as incidence of preoccupation with odor from the body, mouth, armpits, and feet. The scales for pathologic subjective halitosis, olfactory reference syndrome, and social anxiety showed Cronbach’s alpha coefficient values of 0.81, 0.89, and 0.89, respectively.

**Table 1:** Characteristics of PSH ^a^, ORS ^b^ and SA ^c^ and incidence of preoccupation with odors of body parts

	Median (25^th^ percentile, 75^th^ percentile, range)	α coefficient ^d^
Pathologic subjective halitosis ^e^	13 (12, 16; 10–38)	0.81
Olfactory reference syndrome ^e^	14 (10, 19; 7–35)	0.89
Social anxiety ^e^	18 (14, 23; 7–35)	0.89
	N (%)	
Preoccupation with odors of body parts ^f^ (N = 1,360)		
Body odor	738 (54.3)	
Mouth odor	736 (54.1)	
Armpit odor	543 (39.9)	
Foot odor	428 (31.5)	

^a^ Pathologic subjective halitosis

^b^ Olfactory reference syndrome

^c^ Social anxiety

^d^ Cronbach’s alpha coefficient

^e^ Complete data for PSH, ORS, and SA were available for 1,340, 1,346 and 1,350 subjects, respectively

^f^ Participants were able to choose all the options, i.e., body, mouth, armpit, and foot odors

Table 2 shows median values for results of the social anxiety and olfactory reference syndrome scales in 3 levels of the pathologic subjective halitosis scale. The participants were divided based on the results of the pathologic subjective halitosis scale, with the normal group composed of scores from 10 to 13, moderate group of scores from 14 to 21, and the severe group of scores of 22 and higher. Differences were observed among all 3 levels (*P* < 0.001, Kruskal-Wallis test). Those with the highest scores for pathologic subjective halitosis also showed the highest scores for olfactory reference syndrome and social anxiety (Table 2). The differentiation of the scale for olfactory reference syndrome between the normal and moderate groups was 5 points, whereas that between the moderate and severe groups was 10 points. Further, the differentiation of the scale for social anxiety between the normal and moderate groups was 2 points, and that between the moderate and severe groups was 7 points. The results for pathologic subjective halitosis showed that the severe group included only 33 participants.

**Table 2:** Association between the levels of pathologic subjective halitosis, ORS ^a^, and social anxiety

*N* (%)	Pathologic subjective halitosis ^b^			*P* value ^c^
	Normal	Moderate	Severe	
	732 (54.6%)	575 (42.9%)	33 (2.5%)	
Pathologic subjective halitosis	12 ^d^	16	23	<0.001
	(10–13) ^e^	(14–21)	(22–38)	
Olfactory reference syndrome	11	16	26	<0.001
	(7–28)	(7–35)	(11–35)	
Social anxiety	17	19	26	<0.001
	(7–35)	(7–35)	(10–35)	

^a^ Olfactory reference syndrome

^b^ Participants were divided based on the scale of the pathologic subjective halitosis: the normal group included scores of 10–13; the moderate groups included scores of 14–21; the severe group included scores of 22 and over [20]

^c^ Kruskal-Wallis test

^d^ Median

^e^ Score range

Table 3 shows the number and percentage of participants with preoccupations with body part odors among the 3 levels of pathologic subjective halitosis. There were evident differences among the 3 levels for preoccupations with mouth, body, armpit, and foot odor (*P* < 0.05, chi-squared test). The group with severe pathologic subjective halitosis showed higher rates of incidence of preoccupation with mouth and body odor (*P* < 0.05, residual analysis), and the group with moderate pathologic subjective halitosis showed higher rates of incidence of preoccupation with mouth, body, armpit, and foot odor (*P* < 0.05, residual analysis), whereas as those with normal pathologic subjective halitosis showed lower rates of incidence for preoccupation with mouth, body, armpit, and foot odor (*P* < 0.05, residual analysis).

**Table 3:** Relationship between pathologic subjective halitosis and preoccupations with body part odors

*N*	Pathologic subjective halitosis ^a^			*P* value ^b^
	Normal	Moderate	Severe	
	732	575	33	
Mouth odor (○)	337 (46)^c^^▽^	361 (63)^▲^	25 (76)^▲^	<0.001
Mouth odor (−)	395 (54)^▲^	214 (37)^▽^	8 (24)^▽^	
Body odor (○)	359 (49)^▽^	341 (59)^▲^	24 (73)^▲^	<0.001
Body odor (−)	373 (51)^▲^	234 (41)^▽^	9 (27)^▽^	
Armpit odor (○)	261 (36)^▽^	251 (44)^▲^	17 (52)	0.005
Armpit odor (−)	471 (64)^▲^	324 (56)^▽^	16 (48)	
Foot odor (○)	204 (28)^▽^	204 (35)^▲^	11 (33)	0.013
Foot odor (−)	528 (72)^▲^	371 (65)^▽^	22 (67)	

^a^ Participants were divided based on results from the pathologic subjective halitosis scale: the normal group included scores of 10–13; the moderate groups included scores of 14–21; the severe group included scores of 22 and over [20]

^b^ Chi-squared test

^c^ Number (%) of people

^▽^ Residual analysis was used to indicate fewer incidences than the expected value in the same column in the same group of pathological subjective halitosis scores

^▲^ Residual analysis was used to indicate more incidences than the expected value in the same column in the same group of pathological subjective halitosis scores

**Fig. 2:**
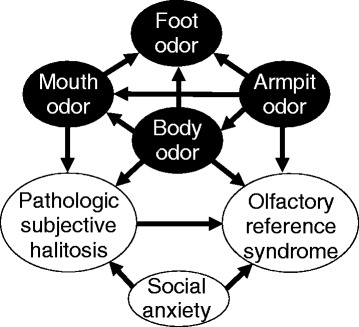
Relationships between pathologic subjective halitosis, olfactory reference syndrome, and preoccupations using Bayesian network analysis

Figure 2 shows our Bayesian network model, which illustrates the causative links between pathologic subjective halitosis, olfactory reference syndrome, social anxiety, and preoccupations with body part odors (mouth odor, body odor, armpit odor, and foot odor). Black circles indicate discrete variables and white circles indicate ordinal variables; causes and effects are indicated by lines and arrowheads, respectively. As the lines (causes) and arrowheads (effects) indicate (Fig. 2), olfactory reference syndrome was caused by pathologic subjective halitosis (i.e., olfactory reference syndrome is an effect of pathologic subjective halitosis). Social anxiety influenced both pathologic subjective halitosis and olfactory reference syndrome. Pathologic subjective halitosis was caused by preoccupations with mouth odor and body odor, and by social anxiety. Olfactory reference syndrome was caused by preoccupations with armpit odor and body odor, as well as pathologic subjective halitosis and social anxiety. The most common causes of pathologic subjective halitosis and olfactory reference syndrome were social anxiety and preoccupation with body odor (Fig. 2). Preoccupation with body odor influenced mouth and foot odor preoccupation, pathologic subjective halitosis, and olfactory reference syndrome; preoccupation with armpit odor influenced mouth, body, and foot odor preoccupation; preoccupations with mouth, body, armpit, and foot odors were reciprocally influential (Fig. 2).

## Discussion

The present study investigated potential causal relationships amongst pathologic subjective halitosis, olfactory reference syndrome, social anxiety, and preoccupations with body part odors. Patients with olfactory reference syndrome are often preoccupied with odors emanating from multiple body parts such as the mouth, body, armpits, and feet [5, 7]. Of these concerns, mouth odor is the most common source of the preoccupation [5], which suggests that olfactory reference syndrome may share features with pathologic subjective halitosis. The present study indicated that the causes that pathologic subjective halitosis and olfactory reference syndrome had in common were social anxiety and preoccupation with body odor (Fig. 2). Given that participants reporting a higher incidence of pathologic subjective halitosis also reported higher social anxiety (Table 2), it is possible that high social anxiety and preoccupation with body odors could induce pathologic subjective halitosis and olfactory reference syndrome.

Participants with pathologic subjective halitosis were preoccupied with not only mouth odor but also body odor (Table 2), which stimulated the others (Fig. 2). This concern with multiple body parts observed in both pathologic subjective halitosis and olfactory reference syndrome may also indicate that the two conditions had mostly overlapping features in the present study.

Seventy-five percent of olfactory reference syndrome patients present with pathologic subjective halitosis complaints [5], suggesting that the latter condition may be present in the former condition. In the present study, olfactory reference syndrome was caused by pathologic subjective halitosis (Fig. 2). Thus, it is possible that olfactory reference syndrome could be triggered by pathologic subjective halitosis. Furthermore, such a relationship might imply that pathologic subjective halitosis could be a subclass of olfactory reference syndrome, which would be consistent with the definition of olfactory reference syndrome [7].

### Analysis of causal relationships

Previous studies have shown that both pathologic subjective halitosis including pseudohalitosis, and olfactory reference syndrome are related to social anxiety disorder [8, 11]; however, the causes of pathologic subjective halitosis and olfactory reference syndrome have historically been difficult to be determined because of unanalyzable causal relationships. Using Bayesian network analysis, the present study was able to elucidate tentative causal relationships between pathologic subjective halitosis, olfactory reference syndrome, social anxiety, and preoccupations with body part odors. Bayesian network is a powerful analysis tool for detecting causal relationships between variables, even in cross-sectional research. Such an approach has not been previously used in the field of pathologic subjective halitosis. The present study is the first to detect possible causes of pathologic subjective halitosis and olfactory reference syndrome.

### Applications in everyday clinical practice

Social anxiety was found to be a cause of both pathologic subjective halitosis and olfactory reference syndrome (Fig. 2); thus, we concluded that treatment strategies for social anxiety disorder may also be applicable as treatment for pathologic subjective halitosis in dental and medical clinics. Generally, the main treatments for social anxiety are cognitive behavioral therapy, management of social anxiety, and brief psychotherapy for social anxiety. However, cognitive behavioral therapy in everyday clinical practice requires psychological specialization and protracted periods of time to change unhelpful patterns in patient cognition. Furthermore, both psychotherapy and pharmacotherapy are used for management of social anxiety [24, 25]. Few dentists are specialists in psychosomatic medicine [26], in which pharmacotherapy is performed for patients with pathologic subjective halitosis [27]. Therefore, it might be difficult to conduct cognitive behavioral therapy and pharmacotherapy for dental practitioners in most dental clinics.

Building a relationship with patients and subsequently making patients reconsider their mouth odor might improve their cognition of mouth odor [28, 29]. Acceptance and support of patients’ mouth odor are important through treatments of pathologic subjective halitosis [28, 29]. Previous studies have employed interviews regarding the patient’s mouth odor and patient-written “Description of impression” about mouth odor to make them reconsider their mouth odor [28, 29]. Such approaches may facilitate patients to notice inconsistency in their cognition of mouth odor.

Pathologic subjective halitosis’ association with social anxiety and olfactory reference syndrome suggests that treatment of patients requires physicians and psychologists. A medical team approach to treat patients with pathologic subjective halitosis that includes collaboration with physicians and psychologists is likely required.

A national qualification for psychologists in medical clinics will soon be established in Japan, and the cooperation of dentists with qualified psychologists for treating pathologic subjective halitosis by management of social anxiety may become a reality in the near future.

At present, patients with pathologic subjective halitosis are often misdiagnosed in general dental and medical clinics [3]. In addition, “doctor shopping,” a term that refers to changing doctors or hospitals without professional referral for the same or similar illness conditions [30], has been frequently observed in patients suffering from halitosis. Dentists should also undergo sufficient training to be able to recognize mental disorders so that patients can be referred to the appropriate specialist [26]. Furthermore, it is important to understand the psychological background of a patient with pathologic subjective halitosis, especially that related to their psychosomatic oral discomfort. Therefore, our findings in this study would lead to a decrease in misdiagnoses of pathologic subjective halitosis, as well as suggestions for new approaches to pathologic subjective halitosis in general practices.

As described above, we found that social anxiety directly influenced pathologic subjective halitosis and olfactory reference syndrome. However, there might still be unknown psychological connections between social anxiety and those pathological conditions. For example, motivation for avoiding rejection [31, 32], body dysmorphic disorder [33], and public self-consciousness [13, 34] might be related to social anxiety. Moreover, body dysmorphic disorder might be associated with olfactory reference syndrome [35]. Psychological characteristics such as motivation for avoiding rejection and body dysmorphic disorder might be potential candidates for elucidating the direct causes of pathologic subjective halitosis and olfactory reference syndrome. Further work will be necessary to clarify such relationships.

### Limitations

A Bayesian network can suggest causal pathways; however, it cannot show the extent of the reliability of pathways. Some pathways have high reliability; others show low reliability in Bayesian networks. The causal pathways shown in our study might not have a high level of reliability due to the cross-sectional nature of the study. Additional analysis is required to explore the underlying psychological processes that may be common to the constructs of pathologic subjective halitosis and social anxiety.

The present participants were community-dwelling female university students attending required health science courses. Although our results demonstrate features of pathologic subjective halitosis and olfactory reference syndrome, they may reflect a potentially higher level of health-consciousness in this particular population. Therefore, the generalizability of our results to a wider variety of individuals should be evaluated with caution.

We did not use organoleptic tests or halitometer measurements to confirm clinical levels of halitosis. Since the scale used for rating pathologic subjective halitosis could not differentiate between subjective and genuine halitosis, some participants may actually have had halitosis. However, a previous report showed that some patients with genuine halitosis also had social anxiety disorder [36], indicating that individuals with any type of halitosis may experience subjective psychological issues such as social anxiety, though their degree may vary. Therefore, the outcomes of the present study are considered to be widely applicable for dental professionals in general practice who treat patients with halitosis.

## Conclusions

The present results suggest that social anxiety is a causal factor of pathologic subjective halitosis and olfactory reference syndrome. Hence, some treatments typically used for social anxiety may be applicable for treating patients with pathologic subjective halitosis, along with collaboration between physicians and psychologists. Furthermore, no crucial differences were found between pathologic subjective halitosis and olfactory reference syndrome.
